# Regulatory monocytes in helminth infections: insights from the modulation during human hookworm infection

**DOI:** 10.1186/s12879-017-2366-0

**Published:** 2017-04-08

**Authors:** Lívia Silva Araújo Passos, Pedro Henrique Gazzinelli-Guimarães, Tiago Antônio de Oliveira Mendes, Ana Clara Gazzinelli Guimarães, Denise da Silveira Lemos, Natasha Delaqua Ricci, Ricardo Gonçalves, Daniella Castanheira Bartholomeu, Ricardo Toshio Fujiwara, Lilian Lacerda Bueno

**Affiliations:** 1 Department of Parasitology, Laboratory of Immunology and Genomics of Parasites, Institute of Biological Sciences, Universidade Federal de Minas Gerais, Belo Horizonte, Brazil; 2 Department of General Pathology, Institute of Biological Sciences, Universidade Federal de Minas Gerais, Belo Horizonte, Brazil; 3 Departament off Biochemistry and Cell Biology, Universidade Federal de Viçosa (UFV), Viçosa, Brazil

## Abstract

**Background:**

While the macrophage polarization is well characterized in helminth infections, the natural heterogeneity of monocytes with multiple cell phenotypes might influence the outcome of neglected diseases, such hookworm infection. Here, we report the profile of monocytes in human hookworm infections as a model to study the regulatory subpopulation of monocytes in helminth infections.

**Methods:**

Blood samples were collected from 19 *Necator americanus*-infected individuals and 13 healthy individuals. Peripheral blood mononuclear cells (PBMCs) were isolated, and immunophenotyping was conducted by flow cytometry. The expressions of genes encoding human nitric oxide synthase (iNOS), interleukin 4 (IL-4), arginase-1 (Arg-1) and glyceraldehyde 3-phosphate dehydrogenase were quantified by qPCR. Plasma levels of IL-4 were determined by sandwich ELISA. Unpaired t-tests or Mann-Whitney tests were used depending on the data distribution.

**Results:**

Hookworm infected individuals (HWI) showed a significant increase in the number of monocytes/mm^3^ (555.2 ± 191.0) compared to that of the non-infected (NI) individuals (120.4 ± 44.7) (*p* < 0.0001). While the frequencies of CD14^+^IL-10^+^ and CD14^+^IL-12^+^ cells were significantly reduced in the HWI compared to NI group (*p* = 0.0289 and *p* < 0.0001, respectively), the ratio between IL-10/IL-12 producing monocytes was significantly elevated in HWI (*p* = 0.0004), indicating the potential regulatory activity of these cells. Measurement of IL-4 levels and gene expression of IL-4 and Arg-1 (highly expressed in alternatively activated macrophages) revealed no significant differences between the NI and HWI groups. Interestingly, individuals from the HWI group had higher expression of the iNOS gene (associated with a regulatory profile) (20.27 ± 2.97) compared to the NI group (11.28 ± 1.18, *p* = 0.0409). Finally, individuals from the HWI group had a significantly higher frequency of CD206^+^CD23^+^IL-10^+^ (7.57 ± 1.96) cells compared to individuals from the NI group (0.35 ± 0.09) (*p* < 0.001), suggesting that activated monocytes are a potential source of regulatory cytokines during hookworm infection.

**Conclusions:**

Natural hookworm infection induces a high frequency of circulating monocytes that present a regulatory profile and promote the downmodulation of the proinflammatory response, which may contribute to prolonged survival of the parasite in the host.

## Background

Monocytes and macrophages are cells of the innate immune system that have well-established roles in the initial response to pathogens and the development of the adaptive immune response as well as the maintenance of tissue homeostasis, wound healing and inflammation [1]. The diverse functions of these cells are related to their ability to adapt to a variety of microenvironments [2]. During inflammatory processes with specific immune responses, the microenvironment of cytokines promotes changes in the physiology of these cells to generate populations with specialized activation programs [3, 4]. Based on Th1/Th2 nomenclature, some authors classify these macrophages as M1 and M2, which are linked to classical and alternative activation profiles [5–8], respectively. However, due to the high plasticity of these cells, it is clear that this classification only includes well-defined populations and does not address subpopulations with transient biochemical and physiological characteristics. In this context, Mosser and Edwards [9] suggested a classification based on the three basic functions of macrophages: a) classically activated macrophages, associated with host defense; b) alternatively activated macrophages, associated with repair of tissue damage; and c) regulatory macrophages associated with immunoregulation.

The classical activation of macrophages is promoted by a combination of signaling through the interferon-γ (IFN-γ) and tumor necrosis factor (TNF) pathways resulting in an increased microbicidal and tumoricidal capacity due to the production of proinflammatory cytokines, superoxide anions and nitrogen radicals [10–12]. In this pathway, arginine is metabolized by inducible nitric oxide synthase (iNOS), which is expressed at high concentrations [13]. Alternatively activated macrophages are generated in response to interleukins 4 and 13 (IL-4, IL-13) from innate or adaptive sources. IL-4 rapidly converts macrophages to a phenotype that promotes tissue healing through the induction of arginase activity. Moreover, macrophage regulators may be generated in response to IL-10 combined with other factors, such as immune complexes, prostaglandins, glucocorticoids, adenosine and apoptotic cells [9, 14, 15]. The combination of these stimuli leads to the development of a population of macrophages that produce high levels of the immunosuppressive cytokine IL-10. Similar to macrophages activated by the classical pathway, regulatory macrophages can also metabolize arginine by nitric oxide synthase [16]. While macrophage polarization is well-characterized in helminth infections [6, 17, 18], the natural heterogeneity of monocytes [1, 19] suggests that multiple cell phenotypes may influence the outcome of the parasitic infection.

Human hookworm infection is a neglected tropical disease caused by the blood-feeding nematodes *Necator americanus*, *Ancylostoma duodenale* and *Ancylostoma ceylanicum* and it is considered the second most important parasitic infection of humans [20]. The presence of a strong immunomodulatory response during chronic hookworm infection is a characteristic feature that allows parasite survival for prolonged periods (5–7 years) in the host intestine [21–23], leading to anemia, malnutrition, growth/cognitive retardation and loss of millions of disability adjusted life-years (DALYs) [24]. Previous studies have showed that human hookworm infection induces peripheral immune responses characterized by increased frequency of regulatory T cells, high levels of circulating IL-10, induction of T lymphocytes apoptosis and modulation of Th17 responses [21, 25–29]. The importance of myeloid cells in the immune modulation during helminth infections was previously shown by the reduced in vitro differentiation of monocyte-derived dendritic cells from hookworm-infected individuals [25]. Indeed, mature DCs from *Necator americanus*-infected individuals also showed significantly down-regulated expression of co-stimulatory and antigen presentation molecules (CD86, CD1a, HLA-ABC and HLA-DR), leading to a reduced ability to induce proliferative responses [25]. Nonetheless, other populations of myeloid cells may also contribute to this robust regulation of immune response and consequent parasite survival. While the number of circulating monocytes is significantly increased during hookworm infection [26], the phenotype and role of these cells in host immunomodulation still remain unclear. The present study aimed to characterize the profile of monocytes in human hookworm infection, providing a model to study the regulatory subpopulation of monocytes in helminth infections, which is still lacking in the literature.

## Methods

### Study population

The present study was conducted in endemic areas for *Necator americanus* in northeast of Minas Gerais State, Brazil. Nineteen volunteers between the ages of 21 and 78 (median age of 47 years; 8 females and 11 males) from areas of moderate *N. americanus* transmission were recruited and presented with light to moderate (up to 840 eggs per gram) intensity of infection. Individuals were selected on the basis of not having any other helminth infection (mono-infection observed after fecal analysis of 12 slides of Kato-Katz thick-smear and Baermann-Moraes technique) and presenting no other medical condition. The presence of *Necator* infection was determined by formalin-ether sedimentation and, when positive, two more stool samples were analyzed by the Kato-Katz fecal thick-smear technique, and parasite load was expressed as eggs per gram of feces (epg) [30]. Seventeen hookworm-naive individuals (10 females and 7 males) were enrolled as healthy non-infected individuals from Belo Horizonte, Minas Gerais State, Brazil, where no transmission occurs. None of these individuals had a history of *Necator* infection and all presented with egg-negative stool (12 slides of Kato-Katz fecal thick smear and Baermann-Moraes technique) and no specific antibodies to *Necator* crude antigen extracts. The geographic areas included in this study are not endemic for tissue-dwelling helminth infections. Furthermore, the nutritional status of non-infected volunteers (controls) was similar to those presented by hookworm-infected individuals as determined by anthropometric measurements. The nutritional status of adults was determined using the absolute body mass index and classified as eutrophic (18.5–24.9 kg/m^2^), underweight (<18.5 kg/m^2^) or overweight (≥25 kg/m^2^) [31]. Approximately 25 mL of blood from *N. americanus* infected and healthy donors was collected in heparinized tubes for separation of peripheral blood mononuclear cells (PBMC) and 4 mL of blood in EDTA tubes for evaluation of the hematological parameters by an automated haematology instrument (Coulter, USA).

### PBMC isolation

Human peripheral blood mononuclear cells (PBMCs) were separated from peripheral blood of *Necator*-infected and healthy donors by gradient centrifugation on Ficoll-Hypaque (GE Healthcare, USA) at room temperature. Cells were then washed twice in RPMI medium (Invitrogen, USA), separated by centrifugation (800 x g for 10 min at 4 °C) and then supplemented with 5% heat-inactivated human AB serum (Sigma, USA), 2 mM of L-glutamine (Sigma, USA), 50 U/mL of penicillin, and 50 g/mL of streptomycin (Invitrogen, USA). Then 1 × 10^6^ cells were cryopreserved in a freezing solution containing 90% SFB (Cultilab) and 10% DMSO (Merck) for use in immunophenotyping assays. These cells were frozen overnight at −80 °C and then transferred to liquid nitrogen. The remaining cells were preserved in RNAlater® solution (Life Technologies, EUA) for use in molecular biology assays.

### PBMC staining and flow cytometry

For monocyte analysis, PBMCs were thawed, transferred to polystyrene tubes and incubated for 4 h with brefeldin A (Sigma, USA). Cell staining was performed using 2 μL of antibodies specific to cell surface markers (anti-CD14 FITC, anti-CD23 FITC, anti-CD206 PE-Cy5; all purchased from Becton Dickinson, USA), with a 30 min incubation at room temperature and protection from light. Following incubation, cells were washed with 2 mL of PBS by centrifugation at 600 x g for 7 min at room temperature. For intracytoplasmatic cytokine staining, cells were submitted to a permeabilization procedure by the addition of 3 mL of Perm buffer (PBS supplemented with 0.5% saponin, Sigma, MO, USA) for 10 min at room temperature. Cells were centrifuged again at 600 x g for 7 min at room temperature. The PBMC were resuspended in 250 μL of Perm buffer and incubated for 30 min with 1 μL anti-cytokine antibodies for IL-10 and IL-12 (Becton Dickinson, CA, USA). After two washing steps, cells were fixed with FACS fixative solution (BD Biosciences, USA) and stored at 4 °C for flow cytometric acquisition. Data were acquired using 10,000 events for each sample. Cell Quest™ software was used for the flow cytometric analysis using a FACSCalibur cytometer (Becton Dickinson, CA, USA).

### Quantitative PCR and determination of cytokine production

In order to evaluate the expression level of genes encoding the human enzymes and cytokines, primers optimized for quantitative PCR were foind in the literature for nitric oxide synthase (iNOS) [32], interleukin 4 (IL-4) [33], arginase-1 (Arg-1) [34] and glyceraldehyde 3-phosphate dehydrogenase (GAPDH) [35]. Glyceraldehyde-3-phosphate dehydrogenase was used as an endogenous control. Total RNA was isolated using a Nucleospin RNA II Kit (Macherey Nagel) from *N. americanus*-infected and healthy donors PBMCs according to the manufacturer’s instructions. The cDNA was prepared using random hexamers and Superscript™ II RnaseH^−^ reverse transcriptase (Invitrogen) according to the manufacturer’s protocol. Primers for all target genes were purchased from Integrated DNA Technologies (IDT) (USA) and used according to the manufacturer’s instruction. The PCR reactions were performed in 96-well plates (MicroAmp®, Applied Biosystems, USA) using 25 μL of following primer, 25 μL, 250 μL of SYBR®-Green (Applied Biosystems, USA) and 100 μL of ultra pure water for PCR (Fermentas, USA) sufficient to prepare a plate. All reactions were performed in triplicate on an ABI Prism 7500 Sequence Detection System (Thermo Scientific, USA). The conditions of thermocycling were 95 °C for 10 min, 40 cycles at 60 °C for 1 min and 95 °C for 15 s. To confirm the specificity of the primers, melt curve analysis of each target was performed after the amplification cycles with 95 °C denaturation for 1 min, 55 °C annealing for 1 min, 80 cycles of 0.5 °C increments for 10 s each, beginning at 55 °C and data collection at each step. Primers were considered specific when the derivative of the melt curve showed only one peak. The expression level of each gene was determined by the relative quantity. Initially, primer efficiency was assessed by using a standard curve for each gene containing five points obtained by serial dilution of a high concentration cDNA sample with the target genes and endogenous control. Efficiency above 95% was considered adequate. The relative expression of each gene in the samples was calculated using the ddCt method [36].

Plasma levels of IL-4 were determined using a sandwich ELISA kit according to the manufacturer’s instructions (R&D Systems). Cytokine concentrations were calculated from the standard curve using 5-parameter curve fitting software (SOFTmax®Pro 5.3, Molecular Devices).

### Statistical analysis

The one-sample Kolmogorov-Smirnov and Shapiro-Wilk tests were used to determine whether variability followed a normal distribution pattern. Unpaired t tests were used to determine the differences of parametric variables between *Necator*-infected individuals and non-infected individuals. Grubb’s test was used to detect the presence of possible outliers. Differences were considered statistically significant when *p* < 0.05.

## Results

### Human hookworm infection promotes increased frequency of monocytes with high production of modulatory cytokines

**Fig. 1:**
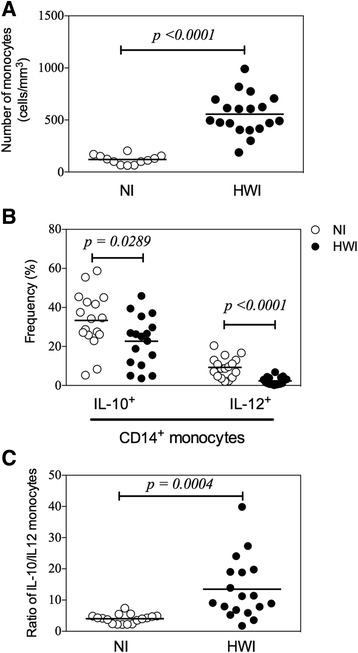
Frequency and cytokine production by monocytes. Comparisons betweeen non-infected (NI) control group and hookworm infected individuals (HWI) for absolute numbers (**a**), frequency of IL-10+ and IL-12+ monocytes (**b**) and ratio of IL-10/IL-12 (**c**) were performed using Student t test. *P* values are indicated on graphs

Initially, flow cytometric analysis of peripheral blood samples from all study participants was performed to investigate differences in the frequency of these cells between the two comparison groups. Hookworm-infected individuals showed a significant increase in the number of monocytes/mm^3^ (555.2 ± 191.0) compared to the NI group (120.4 ± 44.7) (*p* < 0.0001) (Fig. 1a). Since the frequency of monocytes was increased in the HWI group, we further analyzed the profile of these cells and assessed their possible polarization to classically activated or regulatory cells. CD14^+^ cells were identified within PBMCs by their intracellular expression of IL-12 and IL-10, which are key mediators in determining the phenotype of monocytes and macrophages. While the frequency of CD14^+^IL-10^+^ and CD14^+^IL-12^+^ cells was significantly reduced in HWI individuals compared to NI individuals (*p* = 0.0289 and *p* < 0.0001, respectively) (Fig. 1b), the ratio between IL-10/IL-12-producing monocytes was significantly elevated in the HWI group (13.5 ± 2.3) compared to the NI group (4.0 ± 0.4) (Fig. 1c *p* = 0.0004), suggesting the potential regulatory activity of these cells.

### The hookworm infection does not increase IL-4 production by mononuclear cells from peripheral blood

**Fig. 2:**
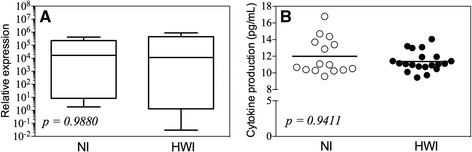
Gene expression and production of IL-4. Comparisons between non-infected (NI) control group and hookworm infected individuals (HWI) for gene expression (**a**) and seric levels (**b**) of IL-4 were performed using Student t test. *P* values are indicated on graphs

Once we demonstrated the predominance of regulatory (IL-10) over pro-inflammatory (IL-12) cytokine producing cells, which minimize the possibility of classical activation, we further evaluated the expression and production of IL-4 by PBMCs of individuals from both groups. When present in sufficient quantities, IL-4 induces the activation of macrophages to the alternative pathway associated with tissue repair function. The assessment of gene expression (Fig. 2a) and production (Fig. 2b) of IL-4 revealed no significant differences between the NI and HWI groups (*p* = 0.9880 and *p* = 0.9411, respectively).

### Hookworm-infected individuals have high iNOS expression in peripheral blood mononuclear cells (PBMCs)

**Fig. 3:**
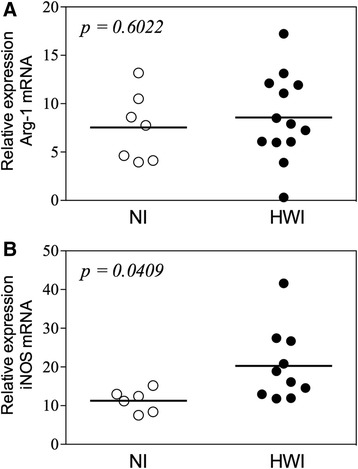
Expression of Arginase-1 and iNOS Comparisons between non-infected (NI) control group and hookworm infected individuals (HWI) for relative expression of Arg-1 (**a**) and iNOS (**b**) were performed using Student t test. *P* values are indicated on graphs

To evaluate the biomarkers associated with metabolism of L-arginine in different populations of monocytes, we assessed the gene expression of arginase-1 (Arg-1) and inducible nitric oxide synthase (iNOS). The evaluation of the Arg-1 gene, which is associated with repair of tissue damage and highly expressed in alternatively activated macrophages, showed no significant difference among the groups (*p* = 0.6022) (Fig. 3a). Interestingly, individuals from the HWI group had higher expression of the iNOS gene (associated with a regulatory profile) (20.27 ± 2.97) compared to the NI group (11.28 ± 1.18, *p* = 0.0409) (Fig. 3b).

### Hookworm-infected individuals display higher frequencies of monocytes expressing regulatory molecules

**Fig. 4:**
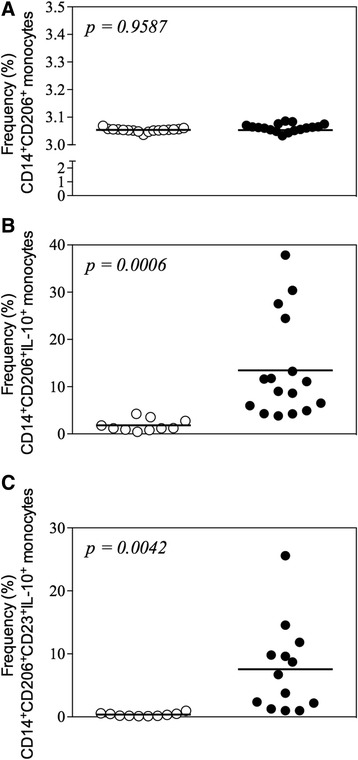
Frequency of CD206^+^ monocytes expressing IL-10 and CD23^+^. **a** Frequency of CD14^+^CD206^+^ (**a**), CD14^+^CD206^+^IL-10^+^ (**b**) and CD14^+^CD206^+^CD23^+^IL-10^+^ monocytes in non-infected (NI) and hookworm infected (HWI) individuals (**c**). Statistical analysis was performed using Student t test. *P* values are indicated on graphs

**Fig. 5:**
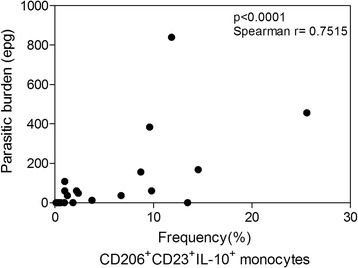
Correlation between frequency of CD14^+^CD206^+^CD23^+^IL-10^+^ monocytes and parasite burden. The correlation analyses were evaluated by Spearman correlation test. A *P*-value <0.05 was considered significant

PBMCs were also evaluated by flow cytometry based on the markers associated with the phenotype of alternatively activated or regulatory monocytes. CD206 (mannose receptor) was used as a marker for the monocyte population, since this molecule is constitutively expressed only by monocytes in the blood and is differentially expressed in “non-classical” (alternatively activated and regulatory) cell populations. Along with the analysis of cell surface CD206, we also assessed the expression of IL-10 by intracytoplasmatic staining. This approach aimed to further confirm that monocytes are a potential source of regulatory cytokines during hookworm infection. While no differences were observed in the frequency of CD206^+^ cells between the HWI and NI groups (Fig. 4a), our results showed that individuals from the HWI group had a significantly higher frequency (*p* = 0.0020) of CD206^+^IL-10^+^ cells (9.20 ± 1.56) compared to individuals of the NI group (1.80 ± 0.40) (Fig. 4b). Finally, positivity for the low affinity IgE receptor CD23 – a surface marker related to cell activation – was also evaluated in double-positive (CD206^+^IL-10^+^) monocytes. Our data showed that individuals from the HWI group had a significantly higher frequency of CD206^+^CD23^+^IL-10^+^ (7.57 ± 1.96) cells compared to individuals from the NI group (0.35 ± 0.09) (*p* < 0.001) (Fig. 4c), indicating the significantly increased number of activated IL-10-producing monocytes during infection. Finally, a correlation analysis between number of monocytes with the regulatory phenotype (CD206^+^CD23^+^IL-10^+^) and parasite load of the individuals evaluated in the study was performed. Interestingly, a strong and positive correlation between infection intensity and regulatory monocytes was observe (Fig. 5).

## Discussion

Monocytes are circulating myeloid immune cells considered the first line of defense against pathogens and primarily associated with differentiation to macrophages and robust cytokine responses. While the impact of these cells in the outcome of helminthic infections has not yet been fully established, the nature of the monocyte response might influence the establishment of the parasite in the host. Similar to previous studies characterizing macrophages [3, 4, 6, 37, 38], we here demonstrate that monocytes present a regulatory phenotype induced by hookworms, which are known to elicit a strong immunomodulatory response during infection [21, 39, 40].

The initial evaluation of circulating monocyte count demonstrated a significant increase in the absolute number of cells (cells/mm^3^) in hookworm-infected individuals (HWI) compared to non-infected individuals, as previously demonstrated in hookworm infections [26]. Such hematological changes are also observed in a wide variety of pathological processes including tuberculosis [41], rheumatoid arthritis [42, 43] and bacterial endocarditis [44]. However, in parasitic infections, the proliferative response of monocytes is commonly observed in protozoal infections such as malaria [45], toxoplasmosis [46] and leishmaniasis [47]. The higher frequency of monocytes during active infections might reflect the consequent increased reactivity of these cells after continuous stimulation promoted by the pathogen, which is not necessarily associated with elimination or control of these bacterial/parasitic infections.

The heterogeneity of function and plasticity of monocytes have been described in parasitic infections [48–50] and clearly demonstrated the differential polarization of these cells as described for macrophages. In our study, phenotypic and molecular parameters were evaluated in circulating monocytes from hookworm-infected individuals to investigate the profile of activation and polarization of these cells. While a reduced frequency of both IL-10^+^ and IL-12^+^ monocytes was observed in the hookworm-infected individuals, regulation of the pro-inflammatory response was prominent and resulted in the higher ratio between IL-10/IL-12 monocytes in the HWI group. Of note, the IL-10/IL-12 ratio is critical in identifying the phenotype of activated macrophages due to differential induction of intracellular signaling pathways that are associated with further cell polarization [2, 8, 9, 51].

While the predominance of the IL-10 response might resemble an M2 profile of activation [52], the basal production of IL-4 in hookworm-infected individuals (similar to that observed in non-infected individuals) suggests the absence of a favorable microenvironment for alternative polarization of the monocytes during hookworm infection. IL-4 stimulation is required for activation of M2 phenotype by cell signaling through the STAT6 pathway (Signal Transducers and Activators of Transcription 6), resulting in increased endocytosis and pinocytosis, inhibition of nitric oxide production due to the activity of arginase, and increased expression of MHC-II and other mediators of tissue remodeling [53].

Polarization of monocytes between alternative and regulatory profiles was also assessed by expression of genes encoding arginase-1 (Arg-1) and the inducible nitric oxide synthase (iNOS). Tissue repair macrophages express high levels of Arg-1, which allows these cells to convert arginine to polyamines and hydroxyproline that directly contribute to extracellular matrix synthesis [54]. Regulatory macrophages are also able to produce nitric oxide (NO) [16], suggesting that these cells may retain some ability to limit intracellular infections despite the fact that this is not their primary physiological role [52]. Our results demonstrate that hookworm infection induces a significant difference in iNOS but not in Arg-1 gene expression between individuals from the HWI and NI groups, which supports the hypothesis of regulatory rather than an alternative profile of activation.

The activation profile was further assessed by the expression of CD206 (mannose receptor), which is a carbohydrate ligand receptor expressed in certain populations of monocytes, macrophages, and dendritic cells in lymphoid tissues and non-vascular endothelium, in constant transit between the plasma membrane and endosomal compartment [55]. Among the functions already described for this receptor are the removal of endogenous molecules, antigen presentation, modulation of cell activation and internalization of collagen [56]. In vitro treatment with IL-4 and IL-13 increased expression levels of CD206, while treatment with IFN-γ and LPS had a negative effect on their expression [57]. Once again, the similar expression of CD206 and IL-4 between infected and control individuals (Figs. 4a and 2, respectively) and the high expression of intracytoplasmic IL-10 within the CD206^+^ monocytes (Fig. 4b) suggest that regulatory monocytes are the predominant circulating myeloid cell population during hookworm infection.

Finally, together with immunophenotypic assays for evaluation of the frequency of CD206^+^IL-10^+^ monocytes, an assessment of the low affinity IgE receptor (CD23/FcεRII) was also performed. The receptor of low affinity IgE (CD23 or FcεRII), which is expressed on the surface of monocytes, neutrophils, eosinophils and B cells, is involved in the regulation of IgE synthesis, cell activation and cytotoxicity [23]. Interestingly, IgE binding to CD23 enhances the antigen presentation by activated cells and results in production and release of nitric oxide [58]. The higher levels of IgE produced during hookworm infection [21, 22] may be associated with a higher frequency of circulating CD23^+^ monocytes (Fig. 4c), which would corroborate the elevated expression of iNOS (Fig. 3b) observed in HWI individuals. Of note, the intensity of the infection is directly associated with a high modulatory capacity of monocytes in human hookworm infection; thus, is evident these cells play an important role in preventing or at least controlling the pathology (Fig. 5).

Limitations of the current study might lie in the restricted number of hookworm mono-infected individuals – a minority among subjects that harbors several parasitic infections at the same time. Moreover, considering that enrolled individuals were asymptomatic and presented low to moderate intensity of infection, *N. americanus*-infected individuals might be considered as long-term chronic patients although the history of previous treatment of multiple exposures to the parasite is unavailable as expected in a cross sectional study. Furthermore, it is important to emphasize the possible limitation of the study for the use of a single reference gene (GAPDH) in the qPCR technique [59]. On the other hand, the use of same conditions was applied to control and test samples and results of qPCR experiment was integrated with others approaches such as flow cytometry to provide more reliable conclusion.

Together, our results suggest that hookworm infection elicits and expand the activation of regulatory monocytes, with downmodulation of IL-12 at a higher extent than IL-10 expression, which might be associated with modulation of the host’s immune response and prolonged survival of the parasite. This circulating myeloid cell population would amplify the downmodulation of immunity observed during hookworm infection as a high frequency of regulatory T cells [29] or apoptotic cells [26] promote a favorable environment for development of regulatory monocytes and/or modulation of monocyte function.

## Conclusion

We conclude that natural hookworm infection induces a high frequency of circulating monocytes that present a regulatory profile and promote the downmodulation of proinflammatory monocyte response. Thus, this myeloid cell population might act cooperatively with other cell populations associated with evasion and regulation during human hookworm infection, and further contribute to the prolonged survival of the parasite in the host.

## Abbreviations

CD: Cluster of differentiation
DALYs: Disability adjusted life-years
IFN: Interferon
IL: Interleukin
PBS: Phosphate buffer saline
Th: T helper
TNF: Tumor necrosis factor

## References

[1] Gordon S, Taylor PR (2005). Monocyte and macrophage heterogeneity. Nat Rev Immunol.

[2] Biswas SK, Mantovani A (2012). Orchestration of metabolism by macrophages. Cell Metab.

[3] Martinez FO, Gordon S, Locati M, Mantovani A (2006). Transcriptional profiling of the human monocyte-to-macrophage differentiation and polarization: new molecules and patterns of gene expression. J Immunol.

[4] Gordon S (2007). The macrophage: past, present and future. Eur J Immunol.

[5] Goerdt S, Orfanos CE (1999). Other functions, other genes: alternative activation of antigen-presenting cells. Immunity.

[6] Gordon S (2003). Alternative activation of macrophages. Nat Rev Immunol.

[7] Mantovani A, Sica A, Locati M (2005). Macrophage polarization comes of age. Immunity.

[8] Mantovani A, Sica A, Sozzani S, Allavena P, Vecchi A, Locati M (2004). The chemokine system in diverse forms of macrophage activation and polarization. Trends Immunol.

[9] Mosser DM, Edwards JP (2008). Exploring the full spectrum of macrophage activation. Nat Rev Immunol.

[10] Mackaness GB (1977). Cellular immunity and the parasite. Adv Exp Med Biol.

[11] Nathan C, Shiloh MU (2000). Reactive oxygen and nitrogen intermediates in the relationship between mammalian hosts and microbial pathogens. Proc Natl Acad Sci U S A.

[12] O'Shea JJ, Murray PJ (2008). Cytokine signaling modules in inflammatory responses. Immunity.

[13] MacMicking J, Xie QW, Nathan C (1997). Nitric oxide and macrophage function. Annu Rev Immunol.

[14] Cohen HB, Briggs KT, Marino JP, Ravid K, Robson SC, Mosser DM (2013). TLR stimulation initiates a CD39-based autoregulatory mechanism that limits macrophage inflammatory responses. Blood.

[15] Sternberg EM (2006). Neural regulation of innate immunity: a coordinated nonspecific host response to pathogens. Nat Rev Immunol.

[16] Edwards JP, Zhang X, Frauwirth KA, Mosser DM (2006). Biochemical and functional characterization of three activated macrophage populations. J Leukoc Biol.

[17] Hussaarts L, Garcia-Tardon N, van Beek L, Heemskerk MM, Haeberlein S, van der Zon GC, Ozir-Fazalalikhan A, Berbee JF, Willems van Dijk K, van Harmelen V (2015). Chronic helminth infection and helminth-derived egg antigens promote adipose tissue M2 macrophages and improve insulin sensitivity in obese mice. FASEB J.

[18] Satoh T, Takeuchi O, Vandenbon A, Yasuda K, Tanaka Y, Kumagai Y, Miyake T, Matsushita K, Okazaki T, Saitoh T (2010). The Jmjd3-Irf4 axis regulates M2 macrophage polarization and host responses against helminth infection. Nat Immunol.

[19] Appleby LJ, Nausch N, Midzi N, Mduluza T, Allen JE, Mutapi F (2013). Sources of heterogeneity in human monocyte subsets. Immunol Lett.

[20] Hotez PJ, Brooker S, Bethony JM, Bottazzi ME, Loukas A, Xiao S (2004). Hookworm infection. N Engl J Med.

[21] Fujiwara RT, Geiger SM, Bethony J, Mendez S (2006). Comparative immunology of human and animal models of hookworm infection. Parasite Immunol.

[22] Loukas A, Prociv P (2001). Immune responses in hookworm infections. Clin Microbiol Rev.

[23] Pritchard DI, Quinnell RJ, Walsh EA (1995). Immunity in humans to *Necator americanus*: IgE, parasite weight and fecundity. Parasite Immunol.

[24] Stephenson LS, Latham MC, Ottesen EA (2000). Malnutrition and parasitic helminth infections. Parasitology.

[25] Fujiwara RT, Cancado GG, Freitas PA, Santiago HC, Massara CL, Dos Santos CO, Correa-Oliveira R, Geiger SM, Bethony J (2009). *Necator americanus* Infection: a possible cause of altered dendritic cell differentiation and eosinophil profile in chronically infected individuals. PLoS Negl Trop Dis.

[26] Gazzinelli-Guimaraes PH, Souza-Fagundes EM, Cancado GG, Martins VG, Dhom-Lemos LC, Ricci ND, Fiuza JA, Bueno LL, Miranda RR, Guatimosim S (2013). Cell apoptosis induced by hookworm antigens: a strategy of immunomodulation. Front Biosci.

[27] Geiger SM, Caldas IR, Mc Glone BE, Campi-Azevedo AC, De Oliveira LM, Brooker S, Diemert D, Correa-Oliveira R, Bethony JM (2007). Stage-specific immune responses in human *Necator americanus* infection. Parasite Immunol.

[28] Geiger SM, Massara CL, Bethony J, Soboslay PT, Correa-Oliveira R (2004). Cellular responses and cytokine production in post-treatment hookworm patients from an endemic area in Brazil. Clin Exp Immunol.

[29] Ricci ND, Fiuza JA, Bueno LL, Cancado GG, Gazzinelli-Guimaraes PH, Martins VG, Matoso LF, de Miranda RR, Geiger SM, Correa-Oliveira R (2011). Induction of CD4(+)CD25(+)FOXP3(+) regulatory T cells during human hookworm infection modulates antigen-mediated lymphocyte proliferation. PLoS Negl Trop Dis.

[30] Katz N, Chaves A, Pellegrino J (1972). A simple device for quantitative stool thick-smear technique in Schistosomiasis mansoni. Rev Inst Med Trop Sao Paulo.

[31] Jardim-Botelho A, Brooker S, Geiger SM, Fleming F, Souza Lopes AC, Diemert DJ, Correa-Oliveira R, Bethony JM (2008). Age patterns in undernutrition and helminth infection in a rural area of Brazil: associations with ascariasis and hookworm. Trop med Int Health.

[32] Jiang ZL, Fletcher NM, Diamond MP, Abu-Soud HM, Saed GM (2009). Hypoxia regulates iNOS expression in human normal peritoneal and adhesion fibroblasts through nuclear factor kappa B activation mechanism. Fertil Steril.

[33] Pouliot P, Turmel V, Gélinas E, Laviolette M, Bissonnette EY (2005). Interleukin-4 production by human alveolar macrophages. Clin Exp Allergy.

[34] Robinson CM, Jung JY, Nau GJ (2012). Interferon-gamma, tumor necrosis factor, and interleukin-18 cooperate to control growth of mycobacterium tuberculosis in human macrophages. Cytokine.

[35] Lai JP, Yang JH, Douglas SD, Wang X, Riedel E (2003). Quantification of CCR5 mRNA in human lymphocytes and macrophages by real-time reverse transcriptase PCR assay. Clin Diagn Lab Immunol.

[36] Pfaffl MW (2001). A new mathematical model for relative quantification in real-time RT-PCR. Nucleic Acids Res.

[37] Goerdt S, Politz O, Schledzewski K, Birk R, Gratchev A, Guillot P, Hakiy N, Klemke CD, Dippel E, Kodelja V (1999). Alternative versus classical activation of macrophages. Pathobiology.

[38] Gordon S (2007). Macrophage heterogeneity and tissue lipids. J Clin Invest.

[39] Brooker S, Bethony J, Hotez PJ (2004). Human hookworm infection in the 21st century. Adv Parasitol.

[40] Loukas A, Constant SL, Bethony JM (2005). Immunobiology of hookworm infection. FEMS Immunol Med Microbiol.

[41] Singh KK, Zhang X, Patibandla AS, Chien P, Laal S (2001). Antigens of mycobacterium tuberculosis expressed during preclinical tuberculosis: serological immunodominance of proteins with repetitive amino acid sequences. Infect Immun.

[42] Dalbeth N, Callan MF (2007). Phenotypic and functional analysis of synovial natural killer cells. Methods Mol Med.

[43] Kojo S, Adachi Y, Keino H, Taniguchi M, Sumida T (2001). Dysfunction of T cell receptor AV24AJ18+, BV11+ double-negative regulatory natural killer T cells in autoimmune diseases. Arthritis Rheum.

[44] Widmer E, Que YA, Entenza JM, Moreillon P (2006). New concepts in the pathophysiology of infective endocarditis. Curr Infect Dis Rep.

[45] Arevalo-Herrera M, Lopez-Perez M, Medina L, Moreno A, Gutierrez JB, Herrera S (2015). Clinical profile of plasmodium falciparum and plasmodium vivax infections in low and unstable malaria transmission settings of Colombia. Malar J.

[46] Wahab MF, El-Gindy IM, Fathy GM (1998). Screening tests for diagnosis of cervical lymphadenopathy presenting as prolonged fever. J Egypt Public Health Assoc.

[47] Bhatia P, Haldar D, Varma N, Marwaha R, Varma S (2011). A case series highlighting the relative frequencies of the common, uncommon and atypical/unusual hematological findings on bone marrow examination in cases of visceral leishmaniasis. Mediterranean J Hematol Infect Dis.

[48] Novais FO, Nguyen BT, Beiting DP, Carvalho LP, Glennie ND, Passos S, Carvalho EM, Scott P (2014). Human classical monocytes control the intracellular stage of Leishmania braziliensis by reactive oxygen species. J Infect Dis.

[49] Semnani RT, Keiser PB, Coulibaly YI, Keita F, Diallo AA, Traore D, Diallo DA, Doumbo OK, Traore SF, Kubofcik J (2006). Filaria-induced monocyte dysfunction and its reversal following treatment. Infect Immun.

[50] Tolouei Semnani R, Moore V, Bennuru S, McDonald-Fleming R, Ganesan S, Cotton R, Anuradha R, Babu S, Nutman TB (2014). Human monocyte subsets at homeostasis and their perturbation in numbers and function in filarial infection. Infect Immun.

[51] Agus DB, Gordon MS, Taylor C, Natale RB, Karlan B, Mendelson DS, Press MF, Allison DE, Sliwkowski MX, Lieberman G (2005). Phase I clinical study of pertuzumab, a novel HER dimerization inhibitor, in patients with advanced cancer. J Clin Oncol.

[52] Fleming BD, Mosser DM (2011). Regulatory macrophages: setting the threshold for therapy. Eur J Immunol.

[53] Martinez FO, Helming L, Gordon S (2009). Alternative activation of macrophages: an immunologic functional perspective. Annu Rev Immunol.

[54] Hesse M, Modolell M, La Flamme AC, Schito M, Fuentes JM, Cheever AW, Pearce EJ, Wynn TA (2001). Differential regulation of nitric oxide synthase-2 and arginase-1 by type 1/type 2 cytokines in vivo: granulomatous pathology is shaped by the pattern of L-arginine metabolism. J Immunol.

[55] Gazi U, Martinez-Pomares L (2009). Influence of the mannose receptor in host immune responses. Immunobiology.

[56] Martinez-Pomares L (2012). The mannose receptor. J Leukoc Biol.

[57] Taylor KS, Counsell CE, Gordon JC, Harris CE (2005). Screening for undiagnosed parkinsonism among older people in general practice. Age Ageing.

[58] Vouldoukis I, Mazier D, Debre P, Mossalayi MD (1995). Nitric oxide and human infectious diseases. Res Immunol.

[59] Kozera B, Rapacz M (2013). Reference genes in real-time PCR. J Appl Genet.

